# Herniotomy for Reducible Inguinal Hernia

**Published:** 1876-09

**Authors:** 


Herniotomy for Reducible Inguinal Hernia.—Mr. — — of Arkansas, had been the subject of hernia for thirteen years, for which he still wears a truss. Coming from a region of country where Gunn's authority in medicine was still supreme, little satisfaction could be obtained in regard to the particulars of his case. Laying aside the truss, an inconsiderable tumor presented itself at the internal abdominal ring and canal, but which did not escape through the external ring. The patient stated that it had never descended into the scrotum. There was also some enlargement of the spermatic cord of this side, probably due to, or increased by, wearing a truss, which he had used for several years.

After consultation with Dr. Buchanan, October 28, 1872, the following operation was performed on the 21st birthday of the patient. A crescentic incision (convexity downwards) was made so as to expose the abdominal canal of the affected side, and the flap dissected up. The little finger of the left hand was now pushed into this canal, carrying before it the loose cellular tissue about the external ring, up to the internal one. Satisfying ourselves that the spermatic cord was safe behind the finger, a canula needle was passed through all the tissues, and the two columns of Ponpart's ligament approximated from the internal to the external ring. Seven stitches were taken, and thus about half of the anterior portion of the whole length of the abdominal canal was permanently closed by silver wire, deposited by the needle.

My idea was, not to draw the stitches very tight, but to secure their ends on perforated shots, so that the wire might be subsequently removed; but, yielding to the opinion of my friend and associate in the case, they were tightened by forceps.

The well-established facts in regard to silver wire being inocuous to the flesh, so that even arteries have been secured, and the peritoneum closed by it with good results, certainly sustains this practice. And while the wound in this instance did not heal by adhesion, something is chargeable to neglect in carrying out the directions in the treatment, so that the patient had developed a crop of boils. Still, at the end of three weeks he left by boat for his distant home; before which, however, he visited, on foot, our State capitol, college, museum, appeared before our class, etc., wearing only a soft cloth over the site of the wound.

Tenth of February, Mr. E. writes us: "I stood the trip home finely, and was very little wearied at any time; my wound is doing very well, though not healing quite so rapidly as I expected. This was only thirty days after the operation.—*Archives of Clinical Surgery.*

